# Negative ANA-IIF in SLE patients: what is beyond?

**DOI:** 10.1007/s10067-023-06577-w

**Published:** 2023-04-04

**Authors:** Hanan Sayed M. Abozaid, Hesham M. Hefny, Esam M. Abualfadl, Mohamad A. Ismail, Amal K. Noreldin, Ahmed N. Nour Eldin, Asmaa M. Goda, Amal H. Ali

**Affiliations:** 1 Rheumatology & Rehabilitation Department, Sohag Faculty of Medicine, Sohag University Hospital, Sohag, 82524 Egypt; 2 Clinical Pathology Department, Sohag University Hospital, Sohag, Egypt; 3 Internal Medicine Department, Sohag University Hospital, Sohag, Egypt; 4 Medical Microbiology and Immunology, Faculty of Medicine, Sohag University, Sohag, Egypt; 5 Medical Microbiology and Immunology, Faculty of Medicine, Aswan University, Aswan, Egypt

**Keywords:** ANA EIA, ANA-IIF, SLE

## Abstract

**Abstract:**

The antinuclear antibody (ANA) test has high sensitivity in diagnosing and classifying systemic lupus erythematosus (SLE).

**Objectives:**

To describe the immunological pattern of SLE patients through investigating specific antinuclear autoantibodies by enzyme dot immunoassay and studying their frequency in both positive and negative ANA indirect immunofluorescence assay (IIF) cases.

**Methods:**

In a cross-sectional study, blood samples from 393 newly diagnosed SLE patients were analyzed using (IIF) on HEp-2 cells and ANA dot immunoassay by automated enzyme immunoassay (EIA) to detect 19 antibodies.

**Results:**

Ninety-one percent of the patients are females; their mean age was 37 ± 12.28. Antinuclear antibody (ANA) was detected by IIF in 82.4% of cases, with 181 (46.1%) speckled and 167 (42.4%) homogeneous ANA patterns. The majority of patients (96%) demonstrated autoantibodies via EIA. Among the ANA-IIF-negative patients, 97.2% demonstrated autoantibodies. There was a significant difference in the frequency of certain autoantibodies between SLE patients with negative and positive ANA-IIF (1.44 0.73, 3.12 2.09, *p* = 0.00) respectively.

**Conclusion:**

The results of analyzing 19 autoantibodies with the ANA staining pattern increased the significance of analyzing the immune profile even if IIF is negative when clinical symptoms strongly suggest SLE diagnosis. Certain autoantibodies may evade staining by the IFA approach while they are present in the patient’s serum, and they may not be detected by the ANA EIA profile if it does not contain that antigenic substrate.

**Table:** 

**Key Points** • *Indirect immunofluorescence on Hep-2 is the conventional method for ANA detection and is regarded as the “gold standard” for testing in clinical practice for SLE*. • *In our study, ANA profile dot enzyme immunoassay (EIA)-based test was performed to evaluate 19 autoantibodies in SLE patients either positive or negative for ANA-IIF*. • *The presence of anti-dsDNA with ANA-IIF-negative serum in 32.4% of SLE patients provides evidence that not all anti-dsDNA antibodies are identified on standard HEp-2 substrates.* • * certain autoantibodies can evade staining by the ANA-IIF method despite being present in the SLE patient’s blood; this supports the ANA profile enzyme dot immunoassay as a more sensitive test.*

## Introduction

Autoimmune diseases (AID) are the result of a self-damaging immune response to its own antigen, affecting 3–9% of the general population. AID are diagnosed based on clinical symptoms and the presence of disease-specific antibodies [1].

Systemic lupus erythematosus (SLE) is a systemic autoimmune illness marked by the development of antinuclear antibodies (ANA) [2] against different intracellular and extracellular components including nucleoproteins, phospholipids, glycoproteins, and glycolipids. The role of these autoantibodies in the pathogenesis of SLE is not fully understood, and only 10% are used in diagnosis [3].

The antinuclear antibodies (ANA) involve both nuclear staining and cytoplasmic and mitotic cell patterns (CMPs); hence, a change in terminology for anti-cellular antibodies (ACAs) is encouraged [4].

The classic method for detecting ANA is indirect fluorescence on Hep-2 (human epithelial cell tumor line) and is regarded as the “gold standard” for testing in clinical practice due to its high sensitivity. This led to the decision to include a positive ANA on HEp-2 cell IFA “or an analogous positive test on other diagnostic platforms” at least once as an entrance requirement for the 2019 EULAR/ACR SLE Classification Criteria [2, 4, 5].

Antinuclear antibodies (ANA) involve both nuclear staining and cytoplasmic and mitotic cell patterns (CMPs); hence, a change in terminology for anti-cellular antibodies is emerging [4]. Other automated tests were developed including solid-phase assays (SPA) to detect autoantibodies in patient’s serum such as enzyme-linked immunosorbent assays (ELISAs) or fluorometric enzyme-linked immunoassays (FEIAs), chemiluminescence immunoassays (CIAs). In addition, there is line or dot immunoassays that enable detection of multiple autoantibodies in the same time [6]. These tests are frequently done for patients with positive ANA test. Their results frequently correlated to disease diagnoses, activity, and complications [7–9].

Certain specialized procedures, such as line immuno assay (LIA), Western blotting, or ELISA, are used to identify specific antibodies for the exact identification of these antibodies [5].

The levels of autoantibodies at the onset of SLE have been divided into three stages. Patients in the first stage, known as normal immunity, are asymptomatic and do not have any measurable autoantibody levels. Benign autoimmunity is the term for the second phase, in which individuals exhibit positive immunological parameters (ANA, anti-Ro, anti-La, and antiphospholipid antibodies) but no immediate clinical signs. Anti-dsDNA, anti-Sm, and anti-RNP are markers of the third phase, which is pathogenic autoimmunity, and are immediately followed by the onset of clinical symptoms of the illness [10]. As a result, the specificity of autoantibodies as well as their existence or absence are useful in the diagnosis and prognosis of diseases [11].

Several studies reported positive autoantibodies by different methods in ANA-IIF assay-negative SLE. In a study by Enis et al., 5% of his patients was negative for ANA-IIF assay and positive for at least one antibody by immunoblot assay [12]. Another study by Gualtierotti reported that 7.4% of their SLE patients were ANA-IIF negative at the beginning of follow-up [13]. A study by Tarazi et al. reported a negative ANA test in a diagnosed patient with cutaneous lupus Erythematosus [14]. Also, Petchiappan et al. reported that 9 of 10 negative ANA-IIF SLE were positive for autoantibodies detected by ELISA [15].

Negative ANA conventional test cannot rule out SLE, and the latest version of the EULAR/ACR criteria supports the use of solid immune assays which can give equivalent results to conventional ANA test [16].

There is limited data about the ANA patterns and its relation to specific autoantibodies and their role in diagnoses of SLE in our locality.

In this study, we aimed to describe the immunological pattern of newly diagnosed Egyptian SLE patients through investigating specific antinuclear autoantibodies using enzyme dot immunoassay and to find out if the frequency of these autoantibodies in SLE patients has positive or negative ANA (IIF) test.

## Material and method

### Study design population

A prospective cross-sectional study included 393 newly diagnosed patients as SLE according to the 2012 Systemic Lupus International Collaborating Clinics (SLICC) classification criteria [17]. These patients had visited the Sohag University Hospital Department of Rheumatology and Rehabilitation in Upper Egypt. Drug-induced lupus and discoid lupus without systemic manifestations were excluded. A complete medical history, a general exam, and evaluations of the skin, cardiovascular, chest, abdomen, neurological, and locomotor systems were obtained. In addition, the age at disease onset (defined as the age at which the first SLE-related symptoms occurred) and disease duration (from the time the first SLE-related symptoms arose until the date of the visit) were recorded; regular laboratory parameters and autoimmune markers were evaluated.

### Ethical approval

The local ethics committee accepted the study protocol with the number IBR#S20-139, and it adheres to the principles stated in the Declaration of Helsinki. Before participating in this study, all patients provided written informed consent.

### Sample collection

Five milliliters of venous blood was withdrawn in a sterile vacutainers tube from each subject under aseptic conditions. Serum was separated using the conventional procedure. The following laboratory investigations were done:

### Antinuclear antibody by indirect immunofluorescence assay on HEp-2 cell

Approximately 20–30 µl of diluted serum (1/40) in phosphate buffered saline (PBS) was placed on fixed HEp-2 cell (Anafluor, DiaSorin, USA) for 30 min at room temperature in a covered damp chamber. Positive and negative controls were run with each test. Slides were rinsed carefully with PBS, and then slides were placed into a Coplin jar filled with PBS for about 10 min. Then, slides were removed from the wash buffer and the excess PBS was drained and the excess PBS was removed using blotting strip.

Approximately 20–30 µl of antihuman immunoglobulin conjugated with fluorescein isothiocyanate (FITC) was dispensed to each well and incubated for an additional 30 min in a dark room to be protected from excessive light. After washing with PBS as before, small drop of mounting media was applied and then a coverslip was placed gently over the slide, and the slides were read using a fluorescence microscope at × 40 power by a technical expert.

ANA by IIF titer of at least 1/80 was regarded as positive. A serum was considered positive if the nuclei of the cell fluoresce with greater or equal intensity than the endpoint reference control well and there is a clearly discernible pattern of fluorescence. Any positive patient specimen was confirmed by repeating the test with twofold dilutions of serum.

The endpoint titer is the serial dilution which best approximates the fluorescent intensity of the endpoint reference control reaction. All positive ANA patterns should be tittered to endpoint dilution to detect possible mixed antinuclear reactions that may not be apparent when interpreting a single screening dilution.

### ANA dot immunoassay by automated enzyme immunoassay EIA

A commercially available dot immunoassay was performed by using Blue Diver Quantrix ANA19 IgG kit (code ANA19Q-24) and Blue Diver Instrument (BDI) (D-tek, Belgium), for the detection of IgG autoantibodies in human sera against nineteen antigens: Nucleosome, dsDNA, Histones, Sm, RNP (68kD/A/C), Sm/RNP, SSA/Ro 60kD, SSA/Ro 52kD, SSB, Scl-70, RNA polymerase III, Ku, PM-Scl 100, Mi-2, Jo-1, CENP-A/B, PCNA, Ribosome P0, and DFS70.

The test is based on a conventional enzyme immunoassay (EIA). Antigens were coated in a microdot-shaped format on a nitrocellulose membrane adhered to a specific plastic support for the test strip. The antigens and controls were all spotted in triplicate.

During the automated testing procedure, the BDI sequentially incubates the strips in the wells of cartridges containing ready-to-use reagents. Initially, the strips were incubated with diluted patient sera. Human antibodies, if present, bind to the specific antigens dotted on the membrane and unbound or excess antibodies are eliminated by washing. The enzyme conjugate binds to antigen–antibody complexes after incubation with goat antibodies conjugated with alkaline phosphatase against human IgG. After rinsing away any excess conjugate, the strips were finally incubated in a substrate solution. The presence of enzyme activity would result in the formation of purple dots. The color intensity was directly related to the concentration of antibody in the sample.

All measured values were totally quantitative when compared to a 6-point, built-in calibration curve, which included a blank control. Multiple types of controls (sample, conjugate, and substrate) were also coated on the strip, and their presence validated the entire testing technique.

Strips were visually examined for discoloration and scanned with a Blue Scan scanner (D-tek, Belgium). Dr. DOT Software was used for the evaluation of the results (D-tek, Belgium). Each strip includes an integrated 6-point standard (calibration) curve with the values 0 (blank), 6, 12, 25, 50, and 100 U/ml; the Dr DOT software measured the mean color intensity of each antigen triplicate and the corresponding quantitative value was calculated from the calibration curve and compared to the predetermined cut-off value to evaluate the result.

The manufacturer recommends a cutoff value of 6 U/ml for the Blue Diver Quantrix ANA19 IgG, while values between 6 and 12 U/ml considered equivocal. A sample was considered positive for a particular antibody if the associated antigen dot value was greater than 12 U/ml.

### Statistical analysis

Using version 20.0 of IBM SPSS Statistics for Windows, data was evaluated. Mean standard deviation, median, and range were used to express quantitative data. Quantitative information was expressed as a percentage and a number. As appropriate, the Chi-square (2) test and Fisher’s exact test were used to compare qualitative variables. As a level of significance, *P* value less than 5% was used for all statistical tests employed in the study.

## Results

### Patient characteristics

The research involved 393 newly diagnosed SLE patients according to the SLICC 2012 classification criteria, 358 (91.1%) are females and 35 (8.9%) are males; the mean age of the patients is 37 ± 12.28, with median 35 and range 26–42 years, mean age at diagnosis 36 ± 11.28 years, and mean disease duration 9 ± 3.6 months.

### Antinuclear antibody by indirect immunofluorescence assay on HEp-2 cell

There were 324 patients (82.4%) positive for ANA and 69 (17.55%) negative; the ANA patterns showed 181 (46.1%) speckled, 167 (42.4%) homogenous, mixed 7 (1.8%), nucleolar 1 (0.3%), mixed pattern included (homogenous and centromere 1 (0.3%), homogenous and nucleolar 2 (0.5%), speckled and homogenous 2 (0.5%), speckled and peripheral 1 (0.3%), and speckled and cytoplasmic ribosomal 1 (0.3%).

### ANA-IIFA titer among study population

The most frequent ANA titer was 1/80 (19.3%) and 1/160 (13.7%), and the maximum titer was 1/10240 (0.3%).

### Comparison between ANA-IIF-positive and ANA-IIF negative SLE patients

ANA-IIF-positive patients showed 13 cases negative for immunoblot assay autoantibodies, 7 of them were homogenous and the other 6 were speckled. Comparison between the ANA +ve patients with the ANA −ve patients has shown significant differences in the frequency of autoantibodies, and the score of the SLICC 2012 criteria (Table 1).

**Table 1:** Comparison between ANA positive and ANA negative SLE patients

	ANA + ve	ANA − ve	*P* value
Age	34.9 ± 12.4	35.9 ± 11.4	0.6
Mean of Abs frequency in each patient	3.12 ± 2.09	1.44 ± 0.73	0.000
SLICC 2012 criteria score	13 ± 5.3	7.8 ± 3.3	0.04

Significant *P* value ≤ 0.05

### Comparison between male and female patients

There was no statistically significant variation between female and male patients in mean age, disease duration, age at diagnosis, SLICC score, and ANA titer, but the significant differences were in the mean number of the associated autoantibodies (2.95, 3.14, *P*= 0.47) respectively. Female patients have significantly higher frequency than males in SS-A/Ro 60kD (44.7%, 26.4%), SS-B/La (15.9%, 2.8%), and SS-A/Ro 52 (33.5%, 2%), while males were significantly higher in nucleosome (45%, 37%), dsDNA (62.8%, 48%), and Sm (34.3%, 13.7).

### ANA profile dot immunoassay tested 19 autoantibodies in all the study participants

The most common findings were for dsDNA in 194 patients (49.4%), nucleosome in 149 (37.9%), and SS-A/Ro 60 in 169 (43%). Twelve autoantibodies were detected in the patients with negative ANA (IIF). Moreover, the incidence of specific autoantibodies varied significantly between SLE patients with negative and positive ANA-IIF (Table 2).

**Table 2:** Autoantibodies frequency detected by ANA dot immunoassay in all SLE groups

Antibodies	ANA − ve (69)	ANA + ve (324)	*P* value	All SLE patients (393)
Nucleosome	13 (18.8%)	136 (41.9%)	0.001	149 (37.9%)
ds-DNA	24 (34.7%)	170 (52.4%)	0.03	194 (49.4%)
Histones	1 (1.4%)	54 (16.6%)	0.018	55 (14%)
Sm	2 (2.8%)	60 (18.5%)	0.04	62 (15.8%)
RNP (68kD/A/C)	1 (1.4%)	69 (21.3%)	0.001	70 (17.8%)
Sm/RNP	2 (2.8%)	79 (24.3%)	0.002	81 (20.6%)
SS-A/Ro 60	9 (13.04)	160 (49.38)	0.002	169 (43%)
SS-A/Ro 52	13 (18.8%)	113 (34.8%)	0.52	126 (32.1%)
SS-B/La	3 (4.3%)	56 (17.3%)	0.217	59 (15%)
Scl70	2 (2.8%)	7 (2.2%)	0.204	9 (2.3%)
RNA-polymerase III	0 (0%)	1 (0.30%)	1	1 (0.3%)
Ku	2 (2.8%)	26 (8.01%)	0.498	28 (7.1%)
PM-Scl 100	0 (0%)	25 (7.7%)	0.151	25 (6.4%)
Mi-2	0 (0%)	1 (0.3%)	1	1 (0.25%)
Jo-1	0 (0%)	1 (0.3%)	1	1 (0.25%)
CENP-A/B	0 (0%)	15 (4.6%)	0.379	15 (3.8%)
PCNA	4 (5.8%)	26 (8.02%)	0.32	30 (7.6%)
Ribosome P0	3 (4.3%)	35 (10.8)	0.236	38 (9.7%)
DFS-70	4 (5.7%)	45 (13.8%)	0.65	49 (12.5%)

*P* value ≤ 0.05 is statistically significant

### The relation between frequency of detected antibodies and each pattern

Negative ANA-IIF showed 34.7% with dsDNA, 30.4% with SS-A/Ro 52, 18.8% with nucleosome, 13.04% with SS-A/Ro 60, and 4.3% with SS-B/La. Homogenous pattern showed 80.8% with ds DNA, 65.9% with nucleosome, 27.5% SS-A/Ro60, 25.1% with histones, 18% with Sm/RNP, and 15% with anti-Sm. Speckled pattern showed 63% with SS-A/Ro 60, 48.6% with SS-A/Ro52, 26% with Sm/RNP, 25.4% with SS-B/La, 24.3% with ds DNA, 20.4% with RNP/68 kD/A/C), and 18.8%with anti-Sm (more details in Table 3).

**Table 3:** Relation between ANA patterns and auto-antibodies

Variables	Homogenous (*n* = 167)	Speckled (*n* = 181)	Mixed (*n* = 7)	Nucleolar (*n* = 1)	Negative (*n* = 69)	Total (*n* = 393)	*P* value
Nucleosome	110 (65.9%)	29 (16%)	2 (28.6%)	0 (0.0%)	13 (18.8)	149 (37.9%)	< 0.001*
dsDNA	135 (80.8%)	44 (24.3%)	3 (42.9%)	0 (0.0%)	24 (34.7%)	194 (49.4%)	< 0.001*
Histones	42 (25.1%)	11 (6.1%)	1 (14.3%)	0 (0.0%)	1 (1.4%)	55 (14%)	< 0.001*
Sm	25 (15%)	34 (18.8%)	2 (28.6%)	0 (0.0%)	2 (2.8%)	62 (15.8%)	0.129
RNP (68 kD/A/C)	29 (17.4%)	37 (20.4%)	3 (42.9%)	0 (0.0%)	1 (1.4%)	70 (17.8%)	0.043*
Sm/RNP	30 (18%)	47 (26%)	2 (28.6%)	0 (0.0%)	2 (2.8%)	81 (20.6%)	0.047*
SS-A/Ro 60	46 (27.5%)	114 (63%)	2 (28.6%)	0 (0.0%)	9 (13.04%)	169 (43%)	< 0.001*
SS-A/Ro52	25 (15%)	88 (48.6%)	3 (42.9%)	0 (0.0%)	21 (30.4)	126 (32.1%)	< 0.001*
SS-B/La	8 (4.8%)	46 (25.4%)	2 (28.6%)	0 (0.0%)	3 (4.3%)	59 (15%)	< 0.001*
Scl70	4 (2.4%)	3 (1.7%)	0 (0.0%)	0 (0.0%)	2 (2.8%)	9 (2.3%)	0.713
RNA-polymerase III	0 (0.0%)	1 (0.6%)	0 (0.0%)	0 (0.0%)	0 (0.0%)	1 (0.3%)	0.882
Ku	12 (7.2%)	15 (8.3%)	0 (0.0%)	0 (0.0%)	1 (2.8%)	28 (7.1%)	0.721
PM-Scl 100	7 (4.2%)	12 (6.6%)	5 (71.4%)	1 (100%)	0 (0.0%)	25 (6.4%)	< 0.001*
Mi-2	1 (0.6%)	0 (0.0%)	0 (0.0%)	0 (0.0%)	0 (0.0%)	1 (0.3%)	0.852
Jo-1	0 (0.0%)	0 (0.0%)	1 (14.3%)	0 (0.0%)	0 (0.0%)	1 (0.3%)	< 0.001*
CENP-A/B	11 (6.6%)	3 (1.7%)	1 (14.3%)	0 (0.0%)	0 (0.0%)	15 (3.8%)	0.052*
PCNA	13 (7.8%)	12 (6.6%)	1 (14.3%)	0 (0.0%)	4 (5.8%)	30 (7.6%)	0.859
Ribosome P0	22 (13.2%)	14 (7.7%)	1 (14.3%)	0 (0.0%)	3 (4.3%)	38 (9.7%)	0.244
DFS-70	21 (12.6%)	24 (13.3%)	0 (0.0%)	0 (0.0%)	4 (5.7%)	49 (12.5%)	0.855

*P* value ≤ 0.05 is statistically significant

## Discussion

As certain antibodies emerge before the onset of clinical symptoms and others are linked to clinical presentations, the identification of autoantibodies in SLE patients can offer insights into pathological processes in diverse tissues and aid in both the diagnosis and monitoring of SLE [5].

In SLE, several autoantibodies have been identified [8]. The categorization criteria of the American College of Rheumatology contain only anti-dsDNA (anti-dsDNA), anti-Smith (anti-Sm), and anti-phospholipid (anti-PL) autoantibodies, even though anti-PL (aPL) autoantibodies are not specific for SLE [9]. While common in SLE, other significant nuclear and cytoplasmic target antigens, such as many ribonuclear proteins (RNP), the RNA binding proteins Ro52 and Ro60, and the 48 kDa protein La, are not unique to SLE. Understanding the connection between particular disease subgroups and autoantibody patterns is a major focus of recent studies.

This study included 393 SLE patients newly diagnosed according to SLICC criteria 2012; indirect immunofluorescence assay (IIF) on HEp-2 cell is done to detect ANA. EIA-based test was performed to evaluate 19 autoantibodies against: nucleosome, double-stranded DNA, histones, Sm, RNP (68kD/A/C), Sm/RNP, SSA/Ro 60kD, SSA/Ro 52kD, SSB, Scl-70, RNA polymerase III, Ku, PM-Scl 100, Mi-2, Jo-1, CENP-A/B, PCNA, Ribosome P0, and DFS70.

Female to male ratio in this study matches with known ratio for SLE. The mean age of our patients at diagnosis was 36±11.28 years, and the mean disease duration was 9±3.6 months. They recently started the medication, so we did not test the relations of medications with the study variables.

Female and male patients did not show significant differences in SLICC score for diagnostic criteria or the mean ANA titer, but the total frequency of the autoantibodies detected through ANA dot immunoassay was significantly more in males than females (3.14, 2.95, *P*= 0.047) respectively, and the higher frequency of SS-A/Ro 60kD (44.7%, 26.4%), SS-B/La (15.9%, 2.8%), and SS-A/Ro 52 (33.5%, 2%) in females than males.

While males were significantly higher in nucleosome (45%, 37%), dsDNA (62.8%, 48%), and Sm (34.3%:13.7), this matches with the findings of Rastin et al. [18] who wanted to assess the clinical and serological characteristics of SLE in Iran. They reported a synchronized decrease in the incidence of anti-SSA and anti-nRNP and a lack of anti-SSB autoantibodies; also, male patients with nephritis have a large rise in dsDNA.

The term antinuclear antibody (ANA) test may not be applicable for a test that includes autoantibodies to antigens in the cytoplasm and mitotic apparatus; the EASI/IUIS recommendations offer the term anti-cellular antibodies instead [19–22]. ICAP publications prefer the name “HEp-2 IIFA” because it includes the whole range of patterns that can be seen when utilizing HEp-2 cells as a substrate [23].

ANA-IIF (82.4%) of the patients were positive with 46.1% showing speckled pattern, 42.4% homogenous, 1.8% mixed pattern, and 0.3% nucleolar; these findings match with other studies [24, 25], while we found 17.5% negative for ANA which is higher than reported by Choi et al. where 6.2% were negative [4]. Another previous study reported that about 2% of systemic lupus erythematosus (SLE) patients does not present ANA positive [26], but our patients are at the early stage of the disease diagnosis while these studies were in a longer disease duration.

The patients with negative ANA-IIF test showed one patient negative with all the tested autoantibodies in ANA profile dot enzyme immunoassay, while the remaining patients have one or more antibodies; the most presented were dsDNA 24 (34.7%), SS-A/Ro52 21 (30.4%), nucleosome 13 (18.8%), SS-A/Ro 60 9 (13.04%), SS-B/La 3 (4.3%), and PCNA 4 (5.8%). Several studies have reported that ANA-negative SLE patients have anti-dsDNA [5, 27, 28]. Additionally, cases of negative antinuclear antibodies, double-stranded DNA antibodies, and positive anti-Ro/SSA antibodies were reported and were associated with severe nephritis. These cases serve as evidence that not all anti-dsDNA antibodies are detected on conventional HEp-2 substrates and that specific dsDNA epitopes may be missed by HEp-2 IIF screening tests [29].

All the study participants and ANA-IIF-positive patients had a 4% negative ANA profile EIA, which is significantly lower than the 27% found by Petchiappan et al. [15]. This difference may be attributed to the fact that our study detected more antibodies that were missed by the ANA profile EIA kits used in Petchiappan et al. Doing an ANA profile EIA directly may save time, but if the specific antigen is not included in the kit, it may not detect the antibodies. In this case, the disease diagnosis and treatment may be delayed. Each test has its own pros and drawbacks.

In the current study, the most common pattern was speckled 181 (46.1%) which shows association with SS-A/Ro 60 (63%), SS-A/Ro52 88 (48.6%), Sm/RNP 47 (26%), and dsDNA 44 (24.3%). Similarly, Menor Almagro et al. reported 69.6% association of speckled pattern with SS-A/Ro 60, SS-A/Ro52, and anti-La [30], followed by homogenous pattern (*N*=167) which was associated with dsDNA135 (80.8%), nucleosome 110 (65.9%), and histones 42 (25.1%). Again, Anis et al. reported that speckled and homogenous were the predominant ANA patterns (56% and 42%, respectively) and they were associated with Sm/RNP, histones, and nucleosomes antibodies [12].

Positive PM-Scl 100 antibodies were associated with nucleolar pattern. Despite reports that anti-PM/Scl antibodies generate a distinctive staining of the nucleoli, detecting anti-PM/Scl seropositivity by IIF is challenging and needs substantial laboratory experience [31].

Limitations of our study were small number of the study group which affected the analysis; also, our patients are newly diagnosed. Follow-up is needed to correlate complications and effect of treatment on the tests used in diagnosis.

## Conclusion

As demonstrated in our study, evaluating 19 autoantibodies’ EIA with the ANA staining pattern increases the usefulness of further investigation using ANA profile dot enzyme immunoassay in the absence of ANA-IIF positivity if the clinical characteristics in a given clinical context are highly suggestive of SLE. The fact that ANA profile enzyme dot immunoassay is more sensitive in the detection of some autoantibodies than ANA-IIF, even when HEP 2 cells are utilized, explains why certain autoantibodies may escape staining by the ANA-IIF method despite still being present in the patient’s serum.

Alternatively, ANA autoantibodies may not be identified by the ANA profile dot immunoassay if the assay does not contain the specific antigenic substrate. Consequently, ANA-IIF is still regarded as the gold standard for screening, but EIA profile is regarded as the confirmatory test; it adds a value in the diagnosis of ANA-IIF-negative cases in correlation to the clinical presentation.

## Data Availability

Available upon request.
